# Professor Ju-Chi Li, one of the pioneers and founders of modern genetics in China

**DOI:** 10.1007/s13238-017-0487-2

**Published:** 2017-10-25

**Authors:** He Zhang

**Affiliations:** 1 Department for the History of Science and Scientific Archaeology, University of Science and Technology of China, Hefei, 230026 China; 2 School of Marxism, Bengbu Medical College, Bengbu, 233030 China

**Figure 1:**
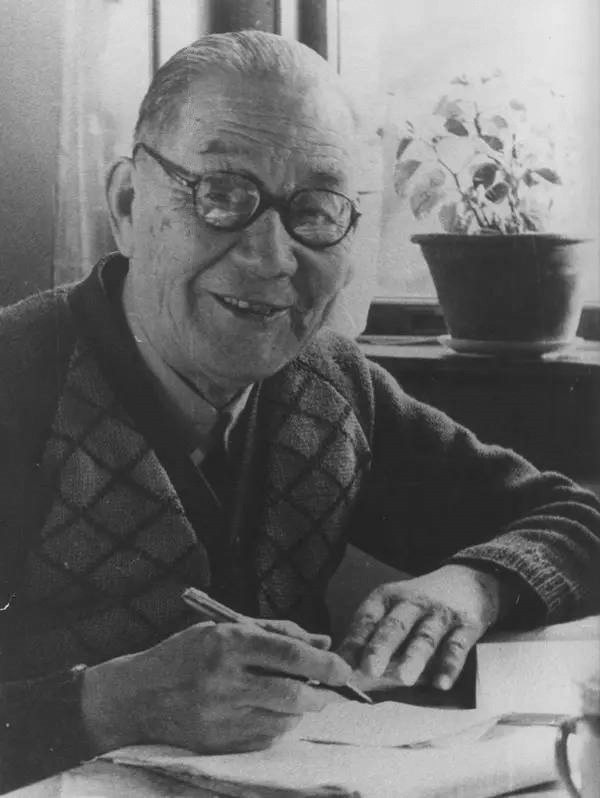
Prof. Ju-Chi Li (1895–1991)

During the year China lost the Sino-Japanese War (1895), he was born in this poor and weak country. In the year the May 4th Movement broke out (1919), he traveled across the oceans to the United States with the ambition of saving the nation by science. It was in the year the Northern Expedition was declared (1926), he returned to his motherland and dedicated himself to science. He was Prof. Ju-Chi Li (Fig. 1), the famous biologist and one of the pioneers and founders of modern genetics in China.

**Figure 2:**
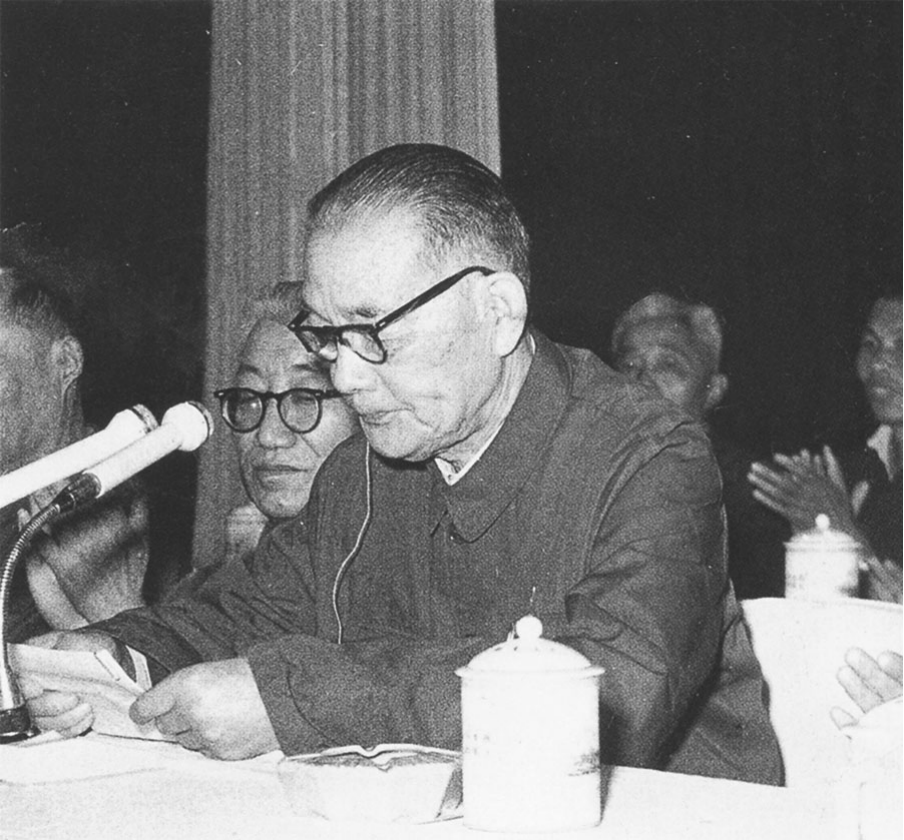
Ju-Chi Li delivering a speech at the founding conference of Genetics Society of China in 1978

Ju-Chi Li was born in Tientsin on March 2nd, 1895. He attended Tsinghua College from 1911 to 1919 before he went to the United States. From 1919 to 1923, he majored in animal husbandry at Purdue University. After graduation, he was admitted to the Department of Zoology at Columbia University, where he focused on the developmental genetics of *Drosophila melanogaster* under the supervision of world famous geneticist Prof. Thomas Hunt Morgan. In 1926, due to his remarkable achievement, he became the first Chinese student to receive a doctor’s degree at Thomas Hunt Morgan’s laboratory. In the same year, he went back to China to teach at Fudan University at Prof. Chiao Tsai’s invitation. The following year, he moved to Yenching University to become a professor in the Department of Biology. In 1952, following the adjustment of colleges and departments in China, he was appointed as a professor in Department of Biology, Peking University until he retired in 1989. Li was promoted to the First Grade Professor in 1956. He was the former director-general of China Zoological Society, the first director-general of Genetics Society of China (Fig. 2) and the editor-in-chief of *Acta Genetica Sinica*.

Li was a pioneer in developmental genetics. As early as 1923, he initiated studies on the effect of chromosome aberrations on development in *Drosophila melanogaster*. Some of the findings in his dissertation for doctor’s degree was published in *Genetics* (Li, 1927). This research article was one of the earliest articles in the field of developmental genetics. It was a dozen years before the American geneticist D. F. Poulson published academic findings in the same field (Poulson, 1940). It was not until the 1960s that developmental genetics was established.

Li had also pioneered the study of chromosome and embryonic development of animals. He took the lead in introducing cytogenetics into China. In 1934, he first reported that *Ascaris megalocephala* having 3 pairs of chromosomes, unlike the classical *Ascaris megalocephala* found in other contries with 1 or 2 pairs of chromosomes, and named it “*Ascaris megalocephala* Trivalens” (Li, 1934). After obtaining new and more abundant experimental materials, in 1937, he announced this important discovery in the top-tier research journal *Science* (Li, 1937), which drew the attention of biologists all over the world. He combined ecology and embryology to study the embryonic developments of *Rana boulengeri*, *Rana nigromaculata*, and *Kaloula borealis* as well as their environmental adaptation, and made outstanding achievements. From 1935 to 1936, he carried out his research on cytogenetics in California Institute of Technology. After his return to China, he introduced the preparative technique of salivary gland chromosome of *Drosophila melanogaster* into China and initiated studies on cytogenetics in China. He and Hsien-Wen Li, Ching-Chun Li, Ching-Hsiung Li together are honored “Four Mars” in genetics area during the Republic era (1912–1949) (Luo, 2010).

It was worth mentioning that Li was invited to attend the Qingdao Meeting on Genetics in August, 1956 (Li and Kang, 2011). During the meeting, he elaborated his point of view and clarified the erroneous criticism of Morganian School along with many other geneticists. He also published an article titled “Talking About Hundred Schools of Thoughts Contend From Genetics” in *Guangming Daily* on April 29th, 1957 based on his speech at the meeting. In this article, he spoke of what he had gained in the meeting and the significance of Hundred Schools of Thoughts Contend for the development of science. Chairman Mao thought highly of this article, changed the title to “The Only Correct Way for the Development of Science” with the original title as its subtitle and even wrote editor’s note for it. It was reprinted in *People*’*s Daily* on May 1st that year and played an active role in promoting the healthy development of science in China (Wu and Dai, 2008).

During his professional career spanned several decades, Li cultivated a large number of talents in the field of genetics to lay the solid foundation for development of modern genetics in China. The eminent geneticist Academician Chia-Chen Tan and animal taxonomist Academician Cheng-Chao Liu finished their master’s dissertations under his supervision. Biologists Tso-Kan Chang, Yin-Chang Chin, Tsu-Ming Lin, and Chao-Te Li were all his brilliant students.

Li accumulated rich experience in his long-term professional career, and accomplished many seminal academic writings. In 1981, his *Principles of Cytogenetics* (Li, 1981) was published and widely used as a general textbook in Chinese colleges and universities before long. At the age of 90, he published his representative work with strenuous effort, which was titled *Developmental Genetics* (Li, 1985a). This book integrated the principal theories of genetics, embryology, and cytology and was regarded as the masterpiece of modern Chinese genetics. In the same year, the book *Selected Papers of Experimental Biology* (Li, 1985b), including forty previously published papers, was delivered. The next year, his *Discussion on Several Problems of Cytogenetics* (Li, 1986) appeared.

In his late years, Li donated his savings to the Genetics Society of China to set up a special fund named “Li Ruqi Animal Genetics Outstanding Paper Award”, which aimed to encourage young scholars to make significant contributions to the development of genetics in China. This was the last great contribution he made for modern genetics in China.
